# The Effects of Preferred Music on Laparoscopic Surgical Performance: A Randomized Crossover Study

**DOI:** 10.1007/s00268-020-05523-0

**Published:** 2020-04-24

**Authors:** Pim Oomens, Victor X. Fu, Vincent E. E. Kleinrensink, Gert-Jan Kleinrensink, Johannes Jeekel

**Affiliations:** 1 Department of Surgery, Erasmus MC, University Medical Center Rotterdam, Doctor Molewaterplein 40, 3015 GD Rotterdam, The Netherlands; 2 Department of Neuroscience, Erasmus MC, University Medical Center Rotterdam, Doctor Molewaterplein 40, 3015 GD Rotterdam, The Netherlands

## Abstract

**Introduction:**

Music can have a positive effect on stress and general task performance. This randomized crossover study assessed the effects of preferred music on laparoscopic surgical performance in a simulated setting.

**Methods:**

Sixty medical students, inexperienced in laparoscopy, were included between June 2018 and November 2018. A randomized, 4-period, 4-sequence, 2-treatment crossover study design was used, with each participant acting as its own control. Participants performed four periods, consisting of five peg transfer tasks each period, on a laparoscopic box trainer: two periods while wearing active noise-cancelling headphones and two periods during music exposure. Participants were randomly allocated to a sequence determining the order of the four periods. The parameters time to task completion, path length and normalized jerk were assessed. Mental workload was assessed using the Surgical Task Load Index questionnaire. Also, heart rate and blood pressure were assessed.

**Results:**

Participants performed the peg transfer task significantly faster [median difference: − 0.81 s (interquartile range, − 3.44–0.69) *p* = 0.037] and handled their instruments significantly more efficient as path length was reduced [median difference, − 52.24 mm (interquartile range, − 196.97–89.81) *p* = 0.019] when exposed to music. Also, mental workload was significantly reduced during music [median difference, − 2.41 (interquartile range, − 7.17–1.83) *p* = 0.021)]. No statistically significant effect was observed on heart rate and blood pressure.

**Conclusion:**

Listening to preferred music improves laparoscopic surgical performance and reduces mental workload in a simulated setting.

**Trial registration:**

Trial registration number: NCT04111679.

## Introduction

In many operating theatres worldwide, music is played. The surgical staff perceives that music can reduce stress and increase efficiency [1, 2]. Much has been published on performance-enhancing effects of music after the observation of a beneficial effect of Mozart’s music on visuospatial task performance [3]. A previous meta-analysis found a small but significant beneficial effect of multiple types of music, not exclusively Mozart’s music, on task performance [4]. Improved task performance could benefit patient outcome. However, earlier studies assessing the effect of recorded music on surgical task performance did not provide conclusive evidence. Two out of nine studies used preferred music, and seven studies used researcher-selected music [5–13]. The available evidence suggests that the participant’s preference could play an essential role in the effects of music on surgical performance [14]. The primary objective of this randomized crossover study is therefore to assess the effect of preferred music on laparoscopic performance in a simulated setting. Secondary objectives are to assess the effect of music on mental workload and vital parameters.

## Materials and methods

This study was approved by the institutional review board of the Erasmus University Medical Center (MEC-2018-1134) and registered at ClinicalTrials.gov (NCT04111679) Written informed consent was obtained from 60 healthy medical students, all inexperienced in laparoscopy, who were included between June 2018 and November 2018.

### Laparoscopic surgical performance assessment

A laparoscopic box trainer was used, which was developed by the skills laboratory of the Erasmus University Medical Center Rotterdam. A shortened version of the peg transfer task was designed to assess laparoscopic surgical performance. The peg transfer task is part of the Fundamentals of Laparoscopic Surgery program, a validated training course mandatory for all surgical residents in the USA [15, 16]. This task consists of moving two colored pegs with grasper forceps using the dominant hand. A sequence of goal positions of the pegs was designated on the screen by colored squares. To assess surgical performance, a Leap Motion device recorded the following parameters: time to task completion (total time used to complete the peg transfer), path length (total distance travelled by the instrument tip during the peg transfer task) and motion smoothness (normalized jerk, i.e., the rate of change in acceleration of instrument tip) [17].

### Mental workload, vital parameters and music listening behavior

Mental workload was assessed using the Surgical Task Load Index (SURG-TLX), a modified version of the National Aeronautics and Space Administration Task Load Index (NASA-TLX) [18]. The SURG-TLX is a weighed 0–100 score based on six subscales that assess mental demands, physical demands, temporal demands, situational stress, task complexity and distractions. After every period, participants completed a SURG-TLX questionnaire. Heart rate and blood pressure were assessed before and immediately after every period, using a Philips© DL8760/15 blood pressure monitor. All participants completed a custom-made demographic questionnaire assessing dexterity, their music listening behavior, whether the participant masters a musical instrument, whether the participant plays or has played video games, and whether the participant listens to music while studying.

### Study design

**Fig. 1:**
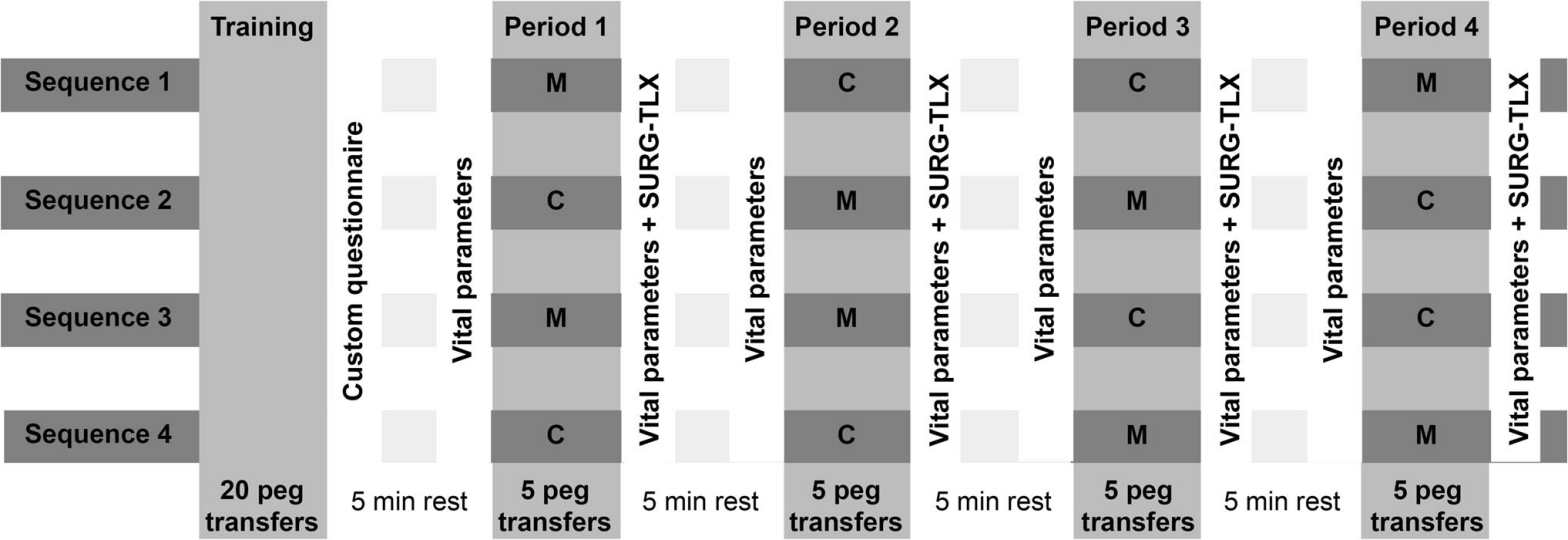
Experiment timeline. The figure provides a timeline of the experiment for each sequence. M = music: participant-selected music via noise-cancelling headphones; C = control: noise-cancelling headphones without music; SURG-TLX: Surgical Task Load Index questionnaire

A 4-period, 4-sequence, 2-treatment crossover design was used in order to reduce a possible learning effect [19]. Participants competed four periods, each consisting of five peg transfer tasks. In total, the peg transfer task was performed 10 times while listening to music selected by the participant (M) and 10 times in silent conditions (C), with every participant serving as their own control. All participants wore noise-cancelling headphones (Bose QuietComfort 35 II) during each period in order to minimize observer bias. Participants were randomly allocated to a sequence that determined the order of the experiment using opaque sealed envelopes. Participants were allowed to rest for 5 min between every period. To get acquainted with the setup and to practice the tasks, all participants completed 20 peg transfers prior to the experiment, as it was observed during the development of the research setup that the learning curve starts to flatten after 20 repetitions. Figure 1 provides an overview of the used sequences as well as a timeline of the experiment.

### Statistical analysis and sample size calculation

Based on the available literature, effect size was estimated at 0.40 [9, 10, 13]. Therefore, a sample size of 52 would be sufficient (*α* = 0.05, *β* = 0.8). The sample size was set at 60 to account for an estimated 10% exclusion rate and to evenly distribute participants over the four sequences in order to maintain a balanced crossover design. Statistical analysis was performed using IBM SPSS Statistics for windows, version 24.0, Armonk, NY: IBM Corp. The normality was assessed using the Kolmogorov–Smirnov test and visually in Q–Q plots. Continuous variable was compared using a paired-samples *T* test if the data were normally distributed or the Wilcoxon signed-rank test if the data were non-normally distributed. Within-subject differences were computed by subtracting the control values from the music values. Within-subject differences of path length, time to task completion and normalized jerk were presented in percentages. Data were presented as median and (interquartile range) if the data were non-normally distributed, or mean ± standard deviation if normally distributed. Between-subgroup differences were calculated using the Student’s *T* test if the data were normally distributed and the Mann–Whitney *U* test if the data were not normally distributed.

## Results

Sixty participants completed the experiment. Demographic characteristics of the included participants are presented in Table 1. The median age was 19 years, and the majority of the participants were first year medical students. Ninety-five percent was right-handed, and the top three favorite musical genres in general were pop, classical and rock music. The musical preference of the participants seemed to depend on the situation, as the top three favorite music genres participants listen to while studying differed, being classical, pop and instrumental music, respectively. Twelve participants never listened to music while studying.

**Table 1:** Demographic characteristics

	Full cohort (*N* = 60)
Age: years (IQR)	19 (18–21)
Sex: male	19 (31.7)
Dexterity: right-handed (%)	57 (95)
Year of study (IQR)	1 (1–3)
Current or previous video game experience	25 (41.7)
Importance of music on a 0–10 scale (IQR)	8 (7–9)
Plays or has played a musical instrument (%)	42 (70)
Top three favorite genres	Pop: 23.90% Classical: 17.90% Rock: 14.10%
Listens to music while studying (%)	48 (80)
Top three favorite genres when studying	Classical: 30.10% Pop: 18.30% Instrumental: 17.20%

### Laparoscopic surgical performance

A statistically significant beneficial effect was observed of music on laparoscopic surgical performance (Table 2). Participants performed the peg transfer task 4.68% (− 4.35–16.59) (*p* = 0.019) faster during music exposure compared to silence. Laparoscopic instrument handling was significantly improved during music exposure as path length was reduced by 6.35% (− 17.15–7.54) (*p* = 0.037). No statistically significant effect of music on normalized jerk was observed (*p* = 0.171).

**Table 2:** Laparoscopic surgical performance data

Full cohort (*N* = 57)	Music	Control	Median of differences	IQR of differences	*p*
Path length (mm)	1141.47 ± 357.42	1216.18 ± 419.81	− 52.24	(− 196.97–89.81)	0.019^a^
Time to task completion (s)	19.16 (16.10–22.68)	19.72 (16.77–24.43)	− 0.81	(− 3.44–0.66)	0.037^b^
Normalized jerk (mm/s^3^)	9595.16 (5885.91–13,934.26)	9538.07 (6394.45–15,631.07)	− 982.12	(− 4635.70–2673.27)	0.171

Continuous variables presented as median (interquartile range) or mean ± standard deviation if the data were normally distributed

Median of differences: median of within-subject differences

IQR of differences: interquartile range of within-subject differences

^a^Paired samples *t* test

^b^Wilcoxon signed-rank

### Mental workload

A statistically significant beneficial effect was observed on the mental workload, as the weighed SURG-TLX score was reduced when participants were exposed to music − 2.41 (− 7.17–1.83) (Table 3). Physical demands were reduced during exposure to music − 2.50 (− 7.50–0.00). Situational stress levels were lower during music exposure − 2.50 (− 11.25–2.50), and the laparoscopic task was perceived as less complex during music exposure − 2.50 (− 7.50–2.50). Music did not reduce distractions or affect mental and temporal demands.

**Table 3:** Mental workload

	Full cohort (*N* = 60)		Within subject	
	Music	Control	*p*	Differences
SURG-TLX	26.00 (14.75–38.00)	28.33 (18.75–42.67)	0.021	− 2.41 (− 7.17–1.83)
Mental demands	20.00 (12.50–35.00)	22.50 (13.75–35.00)	0.160	0.00 (− 6.25–0.00)
Physical demands	15.00 (7.50–18.75)	17.50 (12.50–25.00)	0.003	− 2.50 (− 7.50–0.00)
Temporal demands	22.50 (15.00–52.50)	37.50 (17.50–51.25)	0.156	− 2.50 (− 12.50–7.50)
Situational Stress	12.50 (7.50–31.25)	22.50 (10.00–37.50)	0.005	− 2.50 (− 11.25–2.50)
Complexity	22.50 (12.50–35.00)	27.50 (17.50–35.00)	0.008	− 2.50 (− 7.50–2.50)
Distraction	12.50 (8.75–25.00)	15.00 (10.00–25.00)	0.513	0.00 (− 7.50–0.00)

Surgical Task Load Index (SURG-TLX) is a weighted score comprised of six subscales ranging from 0 to 100 representing general mental workload. Continuous variables presented in medians and (interquartile range)

*P:* Wilcoxon signed-rank test

### Heart rate and blood pressure

No statistically significant effect of music was observed on heart rate, systolic blood pressure and diastolic blood pressure.

## Discussion

To our knowledge, this is the largest published study to date assessing the effect of preferred music on laparoscopic surgical performance in a simulated setting and the first to use participant-preferred music during simulated laparoscopic task performance [14]. Participants performed the laparoscopic task on average 4.68% faster, and their instrument handling was more efficient, as the path length was reduced by 6.35% during exposure to music of their choice. To our knowledge, this is also the first published study that observed a beneficial effect of music on mental workload, as it was significantly reduced during exposure to music. The beneficial effect of music was most profound on situational stress, physical demands and task complexity subscales. Therefore, both laparoscopic surgical task performance and the subjective experience of the participants were significantly improved during music exposure.

In surveys assessing healthcare provider’s attitudes toward music in the operating room, the operating room staff reported that music made them feel less stressed and a majority considered music not distracting [2, 20]. In the present study using a simulated setting, we observed similar effects. Music reduced the situational stress and was not found to be distracting. In fact, a majority of our cohort experienced music as less distracting than silence. The present observations using participant-preferred music confirm earlier observations by Miskovic et al. [9], who observed a trend toward a statistically significant beneficial effect on laparoscopic surgical performance when participants considered the researcher-selected music as pleasant. Our results confirm earlier suggestions that participant-preferred music improves simulated surgical performance [21]. Perioperative music has been observed to reduce stress in patients undergoing surgery [22]. We observed comparable effects during laparoscopic surgical task performance when participants were exposed to music in situational stress. High levels of stress and anxiety in the operating room are associated with impaired surgical performance, team performance and an increased risk of adverse events; music might help to reduce these stress levels [22–24].

Preferred music reduced subjective physical demands during laparoscopic performance. This could be of importance, as surgery is a physically demanding task. Musculoskeletal disease is highly prevalent in surgeons, often leading to a leave of absence, practice restriction or even early retirement [25, 26]. High levels of workload and perceived physical demands have been associated with the incidence of musculoskeletal disease in healthcare workers [27–30].

This study has strengths and limitations. Our sample size is large compared to earlier studies that assessed the effect of music on surgical performance [5–13]. Our crossover design is well balanced and minimizes possible learning effects [19]. To further reduce this effect, all participants practiced the laparoscopic task prior to the experiment to the point where it has been observed that the learning curve starts to flatten during development of our research setup. Our study assessed laparoscopic surgical performance in a simulated setting, which has been observed to translate to, and correlates with real-world surgical performance [31–34]. Music was not considered to be distracting and positively affected laparoscopic surgical task. However, music during surgery has been reported to impair team communication, which could not be assessed in our experimental setup. It cannot be ruled out that the beneficial effects we observed can be attributed to auditory stimulation and not music per se, given that the control condition was silence. As it has been reported that noise cancellation could induce anxiety in patients [35], it could even be possible that we observed an adverse effect of silence instead of a beneficial effect of music. Furthermore, in a live operating environment, it is hardly ever silent in the operating room [36]. It is unclear how music would affect laparoscopic performance in a noisy environment such as the operating theatre or when controlling for another form of auditory stimulation. However, given that music is generally well liked and prevalently played during surgery, we do not believe it clinically relevant to assess the effects of other forms of auditory stimulation for surgical task performance, except recorded operating room noise. The study population consisted of medical students who were inexperienced in laparoscopy, performing a relatively simple laparoscopic peg transfer task, as it has been reported that more experienced surgeons can more effectively block out noise and music. Also, several practical barriers would hinder the inclusion of a significant number of surgeons for laparoscopic task performance in a simulated setting, like time constraints due to busy schedules. How music would affect performance and mental workload while performing more complex surgical tasks remains unclear. Unfortunately, we could not identify any personal factors that enhance or diminish the effects of music on laparoscopic surgical performance, due to our sample size.

Surgical residents report that the largest barrier to attend simulation-based training is a lack of free time [37]. Implementing music interventions could assist surgical residents in completing their mandatory training modules more quickly and efficiently [15]. In our opinion, future research should focus on the effects of music while controlling for the noisy environment of the operating theater and the effects of music on laparoscopic tasks with a higher level of complexity. A follow-up study is currently being conducted investigating aforementioned fields of interests (Dutch Trial Register (Trial NL7961) www.trialregister.nl/trial/7961). Also, it would be interesting to investigate the effect of music on surgical performance in more experienced surgeons, as it is hypothesized that they block out distracting noises more effectively [10].

## Conclusion

Preferred music improves laparoscopic surgical performance and reduces mental workload in a simulated setting.

## References

[1] Makama JG, Ameh EA, Eguma SA (2010). Music in the operating theatre: opinions of staff and patients of a Nigerian teaching hospital. Afr Health Sci.

[2] Ullmann Y, Fodor L, Schwarzberg I (2008). The sounds of music in the operating room. Injury.

[3] Rauscher FH, Shaw GL, Ky KN (1993). Music and spatial task performance. Nature.

[4] Pietschnig J, Voracek M, Formann AK (2010). Mozart effect-Shmozart effect: a meta-analysis. Intelligence.

[5] Conrad C, Konuk Y, Werner P (2010). The effect of defined auditory conditions versus mental loading on the laparoscopic motor skill performance of experts. Surg Endosc Interv Tech.

[6] Conrad C, Konuk Y, Werner PD (2012). A quality improvement study on avoidable stressors and countermeasures affecting surgical motor performance and learning. Ann Surg.

[7] Kyrillos R, Caissie M (2017). Effect of music on surgical skill during simulated intraocular surgery. Can J Ophthalmol.

[8] Lies SR, Zhang AY (2015). Prospective randomized study of the effect of music on the efficiency of surgical closures. Aesthet Surg J.

[9] Miskovic D, Rosenthal R, Zingg U (2008). Randomized controlled trial investigating the effect of music on the virtual reality laparoscopic learning performance of novice surgeons. Surg Endosc Interv Tech.

[10] Moorthy K, Munz Y, Undre S (2004). Objective evaluation of the effect of noise on the performance of a complex laparoscopic task. Surgery.

[11] Shakir A, Chattopadhyay A, Paek LS (2017). The effects of music on microsurgical technique and performance: a motion analysis study. Ann Plast Surg.

[12] Siu KC, Suh IH, Mukherjee M (2010). The effect of music on robot-assisted laparoscopic surgical performance. Surg Innov.

[13] Wiseman MC (2013). The mozart effect on task performance in a laparoscopic surgical simulator. Surg Innov.

[14] Oomens P, Fu VX, Kleinrensink GJ (2019). The effect of music on simulated surgical performance: a systematic review. Surg Endosc.

[15] Hafford ML, Van Sickle KR, Willis RE (2013). Ensuring competency: are fundamentals of laparoscopic surgery training and certification necessary for practicing surgeons and operating room personnel?. Surg Endosc.

[16] Surgeons SoAGaE Fundamentals of Laparoscopic Skills (FLS) Program, 2018

[17] Ghasemloonia A, Maddahi Y, Zareinia K (2017). Surgical skill assessment using motion quality and smoothness. J Surg Educ.

[18] Wilson MR, Poolton JM, Malhotra N (2011). Development and validation of a surgical workload measure: the surgery task load index (SURG-TLX). World J Surg.

[19] Hedayat AS, Stufken J (2003). Optimal and efficient crossover designs under different assumptions about the carryover effects. J Biopharm Stat.

[20] Yamasaki A, Mise Y, Mise Y (2016). Musical preference correlates closely to professional roles and specialties in operating room: a multicenter cross-sectional cohort study with 672 participants. Surgery.

[21] Oomens P, Fu VX, Kleinrensink GJ (2019). The effect of music on simulated surgical performance: a systematic review. Surg Endosc.

[22] Fu VX, Oomens P, Sneiders D (2019). The effect of perioperative music on the stress response to surgery: a meta-analysis. J Surg Res.

[23] Arora S, Sevdalis N, Nestel D (2010). The impact of stress on surgical performance: a systematic review of the literature. Surgery.

[24] Chrouser KL, Xu J, Hallbeck S (2018). The influence of stress responses on surgical performance and outcomes: literature review and the development of the surgical stress effects (SSE) framework. Am J Surg.

[25] Stucky CH, Cromwell KD, Voss RK (2018). Surgeon symptoms, strain, and selections: systematic review and meta-analysis of surgical ergonomics. Ann Med Surg (Lond).

[26] Epstein S, Sparer EH, Tran BN (2018). Prevalence of work-related musculoskeletal disorders among surgeons and interventionalists: a systematic review and meta-analysis. JAMA Surg.

[27] Choobineh A, Movahed M, Tabatabaie SH (2010). Perceived demands and musculoskeletal disorders in operating room nurses of Shiraz city hospitals. Ind Health.

[28] Kim IH, Geiger-Brown J, Trinkoff A (2010). Physically demanding workloads and the risks of musculoskeletal disorders in homecare workers in the USA. Health Soc Care Community.

[29] Passier L, McPhail S (2011). Work related musculoskeletal disorders amongst therapists in physically demanding roles: qualitative analysis of risk factors and strategies for prevention. BMC Musculoskelet Disord.

[30] Trinkoff AM, Lipscomb JA, Geiger-Brown J (2003). Perceived physical demands and reported musculoskeletal problems in registered nurses. Am J Prev Med.

[31] Nagendran M, Gurusamy KS, Aggarwal R, et al (2013) Virtual reality training for surgical trainees in laparoscopic surgery. Cochrane Database Syst Rev CD006575 10.1002/14651858.CD006575.pub3 PMC7388923 23980026

[32] Nagendran M, Toon CD, Davidson BR, et al (2014) Laparoscopic surgical box model training for surgical trainees with no prior laparoscopic experience. Cochrane Database Syst Rev CD010479 10.1002/14651858.CD010479.pub2 PMC10875404 24442763

[33] Sroka G, Feldman LS, Vassiliou MC (2010). Fundamentals of laparoscopic surgery simulator training to proficiency improves laparoscopic performance in the operating room: a randomized controlled trial. Am J Surg.

[34] Sturm LP, Windsor JA, Cosman PH (2008). A systematic review of skills transfer after surgical simulation training. Ann Surg.

[35] Ames N, Shuford R, Yang L (2017). Music listening among postoperative patients in the intensive care unit: a randomized controlled trial with mixed-methods analysis. Integr Med Insights.

[36] Katz JD (2014). Noise in the operating room. Anesthesiology.

[37] Gostlow H, Marlow N, Babidge W (2017). Systematic review of voluntary participation in simulation-based laparoscopic skills training: motivators and barriers for surgical trainee attendance. J Surg Educ.

